# Consensus tissue domain detection in spatial omics data using multiplex image labeling with regional morphology (MILWRM)

**DOI:** 10.1038/s42003-024-06281-8

**Published:** 2024-10-30

**Authors:** Harsimran Kaur, Cody N. Heiser, Eliot T. McKinley, Lissa Ventura-Antunes, Coleman R. Harris, Joseph T. Roland, Melissa A. Farrow, Hilary J. Selden, Ellie L. Pingry, John F. Moore, Lauren I. R. Ehrlich, Martha J. Shrubsole, Jeffrey M. Spraggins, Robert J. Coffey, Ken S. Lau, Simon N. Vandekar

**Affiliations:** 1 Epithelial Biology Center, Vanderbilt University Medical Center, Nashville, TN USA; 2 Program in Chemical and Physical Biology, Vanderbilt University School of Medicine, Nashville, TN USA; 3 Department of Cell and Developmental Biology, Vanderbilt University School of Medicine, Nashville, TN USA; 4 Department of Neurology, Vanderbilt University Medical Center, Nashville, TN USA; 5 Department of Biostatistics, Vanderbilt University Medical Center, Nashville, TN USA; 6 Center for Quantitative Sciences, Vanderbilt University School of Medicine, Nashville, TN USA; 7 Department of Surgery, Vanderbilt University Medical Center, Nashville, TN USA; 8 Mass Spectrometry Research Center, Vanderbilt University School of Medicine, Nashville, TN USA; 9 Department of Biochemistry, Vanderbilt University School of Medicine, Nashville, TN USA; 10 Department of Molecular Biosciences, The University of Texas at Austin, Austin, TX USA; 11 Department of Medicine, Division of Epidemiology, Vanderbilt Epidemiology Center, Vanderbilt University Medical Center, Nashville, TN USA; 12 Vanderbilt-Ingram Cancer Center, Nashville, TN USA; 13 Department of Pathology, Microbiology and Immunology, Vanderbilt University Medical Center, Nashville, TN USA; 14 Division of Gastroenterology, Hepatology and Nutrition, Department of Medicine, Vanderbilt University Medical Center, Nashville, TN USA

**Keywords:** Image processing, Software

## Abstract

Spatially resolved molecular assays provide high dimensional genetic, transcriptomic, proteomic, and epigenetic information in situ and at various resolutions. Pairing these data across modalities with histological features enables powerful studies of tissue pathology in the context of an intact microenvironment and tissue structure. Increasing dimensions across molecular analytes and samples require new data science approaches to functionally annotate spatially resolved molecular data. A specific challenge is data-driven cross-sample domain detection that allows for analysis within and between consensus tissue compartments across high volumes of multiplex datasets stemming from tissue atlasing efforts. Here, we present MILWRM (multiplex image labeling with regional morphology)—a Python package for rapid, multi-scale tissue domain detection and annotation at the image- or spot-level. We demonstrate MILWRM’s utility in identifying histologically distinct compartments in human colonic polyps, lymph nodes, mouse kidney, and mouse brain slices through spatially-informed clustering in two different spatial data modalities from different platforms. We used tissue domains detected in human colonic polyps to elucidate the molecular distinction between polyp subtypes, and explored the ability of MILWRM to identify anatomical regions of the brain tissue and their respective distinct molecular profiles.

## Abstract (web summary)

MILWRM is a Python package that can perform tissue domain detection on spatial transcriptomics (ST) and multiplex immunofluorescence (mIF) data across multiple specimens.

## Introduction

The advent of spatially resolved molecular assays has enabled access to high dimensional genetic, transcriptomic, proteomic, and even epigenetic information in situ while preserving the spatial information lost in single-cell or bulk molecular assays^1–4^. Spatially resolved data can provide powerful insight into interactions between cell types, progressive changes in tissue architecture in diseases such as cancer, or interactions between different structures in tissue such as lymphoid follicles and blood vessels^5–7^. Biological insights can be derived from recurring spatial patterns extracted using quantitative analysis of spatial data.

Many current methods to detect spatial domains attempt to complement single-cell analyses, essentially taking a bottom-up approach to reconstruct tissue domains, architectures, and communities from individual cells. In general, segmentation can identify individual cells from high-dimensional imaging data. Cellular segmentation and annotation are the most challenging step in this kind of approach. While numerous methods are available for cellular segmentation^8,9^ and annotation^10^, it remains an error-prone task that introduces bias due to the potential failure of segmenting certain cell shapes or types. Methods such as unsupervised discovery of tissue architecture with graphs^11^ and SpatialLDA^12^ are examples of two such algorithms that take segmented cell data as input to identify microanatomical structures or tissue microenvironments. However, results generated from these algorithms are highly dependent on the segmentation algorithm used for preprocessing.

Widely used lower-resolution imaging data such as spatial transcriptomics (ST) data are analyzed using cellular deconvolution algorithms to approximate single-cell composition. Most of these algorithms require a parallel single-cell dataset for use as reference^13,14^. Different cell types are then arranged into interaction networks based on their spatial distributions and/or molecular interactions, and these networks are assembled into larger spatial structures that identify tissue- or organ-level domains. This type of analysis has been used for identifying cellular communities in various cancer types associated with patient prognosis^15–18^.

Another perspective comes from the pathology field, where spatial domains and architectures are first identified, followed by instances of cell identification by morphology, which is known as the top–down approach^19^. Since this approach focuses directly on pixel-level information instead of reconstruction from single-cell data, it can identify both extracellular structures and cellular communities over a range of micro- and macro-scales. Pixel-based analysis reduces bias introduced by single-cell segmentation and allows for the implementation of modern artificial intelligence to be applied to multiplex tissue data^20–22^.

Various methods are already available for top–down pixel-based spatial domain detection from ST data^23–28^. However, many of them lack the scalability to work across samples to identify consensus domains. Instead, they identify regional domains that are sample-specific or confounded by batch effects. While manual curation of sample-specific domains is routinely performed, this process again introduces biases and does not scale to many samples. Furthermore, these tools are not transferable to other data modalities and there is a notable paucity of pixel-based tissue domain (TD) identification algorithms for imaging data. The basis of pathological identification of diseases hinges on discerning recurring morphologies, shapes, and color patterns within tissues across patient cases. Extension of this concept to high dimensional spatial assays is akin to identifying biologically relevant spatial domains that exhibit consensus across samples. Here, we present multiplex image labeling with regional morphology (MILWRM) which is a top–down algorithm designed specifically for consensus TD characterization across large data sets from multiplex immunofluorescence (mIF) and ST modalities with potentially differing orientations and resolutions.

## Results

### The MILWRM pipeline generates consensus tissue domains across specimens

**Fig. 1:**
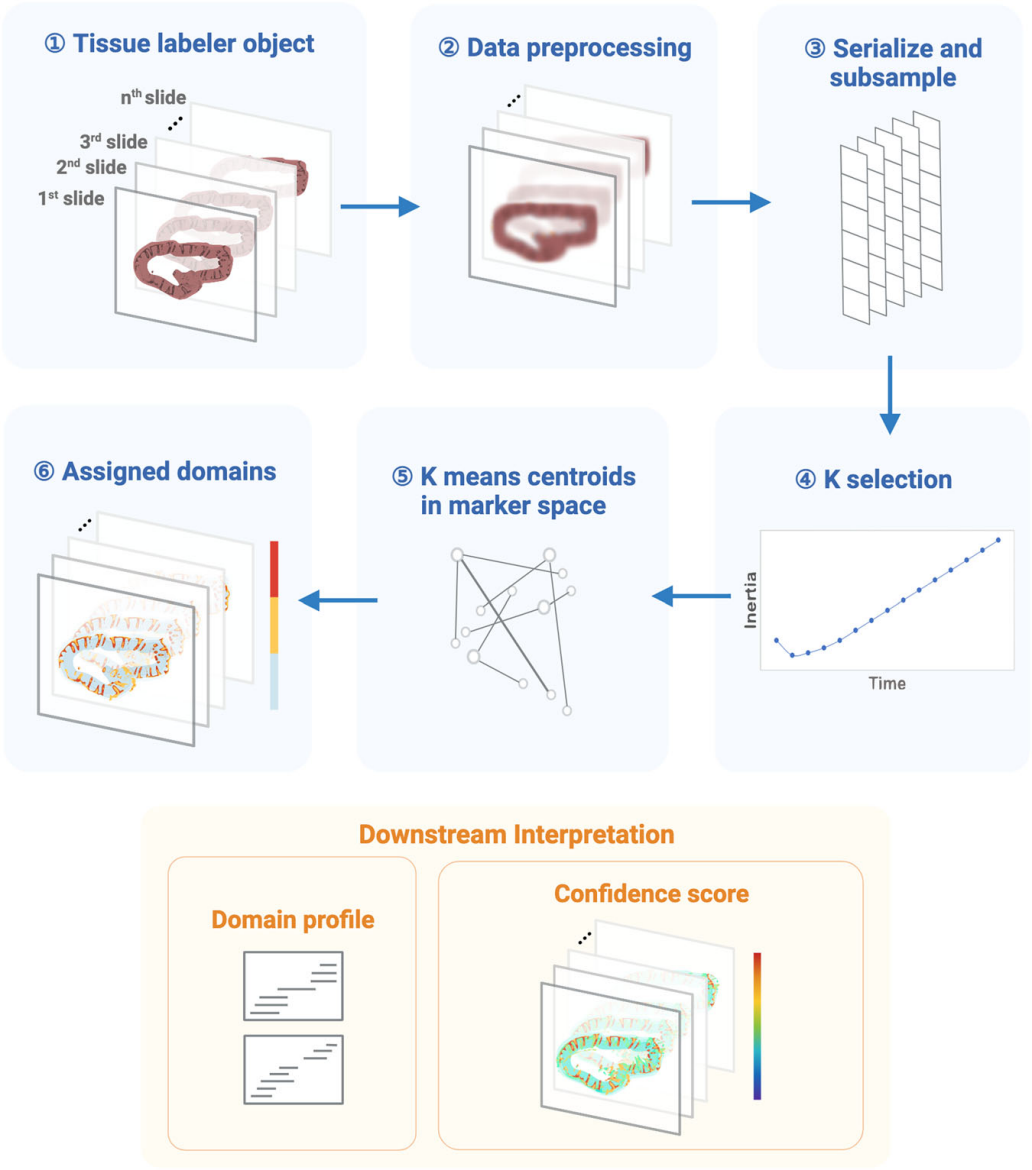
The workflow of the MILWRM pipeline. MILWRM begins with constructing a tissue labeler object from all the sample slides that undergo data preprocessing, serialization, and subsampling to create a randomly subsampled dataset used for *k*-means model construction. This subsampled data is used to find an optimal number of tissue domains, and *k*-selection using the adjusted inertia method. Finally, a *k*-means model is constructed, and each pixel is assigned a TD. Each TD has a distinct domain profile describing its molecular features. MILWRM also provides quality control metrics such as confidence scores (created with BioRender.com).

MILWRM is a cross-modal, pixel- or spot-level algorithm that identifies consensus tissue domains across samples with spatially resolved molecular data (e.g., mIF and ST) that can be applied to multiple specimens, unlike most spatial analysis algorithms. Because it is a pixel-based algorithm, it bypasses bias introduced by single-cell segmentation. The MILWRM pipeline can be broadly categorized into three major steps: data preprocessing (Fig. 1; 1–3), TD identification (Fig. 1; 4–6), and TD analysis (Fig. 1; downstream interpretation). To generalize pixel neighborhood information across batches, data preprocessing incorporates down-sampling, normalization, data smoothing, and dimensionality reduction. Preprocessing steps differ slightly for mIF and ST (see “Methods”). After preprocessing, tissue domains are identified using *k*-means clustering by randomly subsampling pixels uniformly within the tissue mask data with a proportion of 0.2 from each sample (see “Methods”). *k*-Means was selected among other clustering algorithms due to its computational efficiency which allows the analysis to be performed across all slide subsets simultaneously after *Z*-normalization and allows the resulting model to be applied to the full dataset. The number of tissue domains can simply be given as input or adjusted by inertia analysis in an unsupervised manner^29^. Each pixel is assigned a TD based on the nearest centroid which is determined in the original data by using the parameters estimated from the *Z*-normalization in the subset. Domain profiles are calculated by MILWRM from the initial feature space to molecularly describe each TD, which is useful for downstream annotation. Finally, MILWRM computes a variety of metrics to assess the quality of identified tissue domains (see “Methods”). Overall, MILWRM is a comprehensive, easy-to-use pipeline for TD detection, providing interpretable results for biological analysis and quality assessment.

### Tissue domain (TD) detection in multiple mIF datasets from various tissues and platforms

**Fig. 2:**
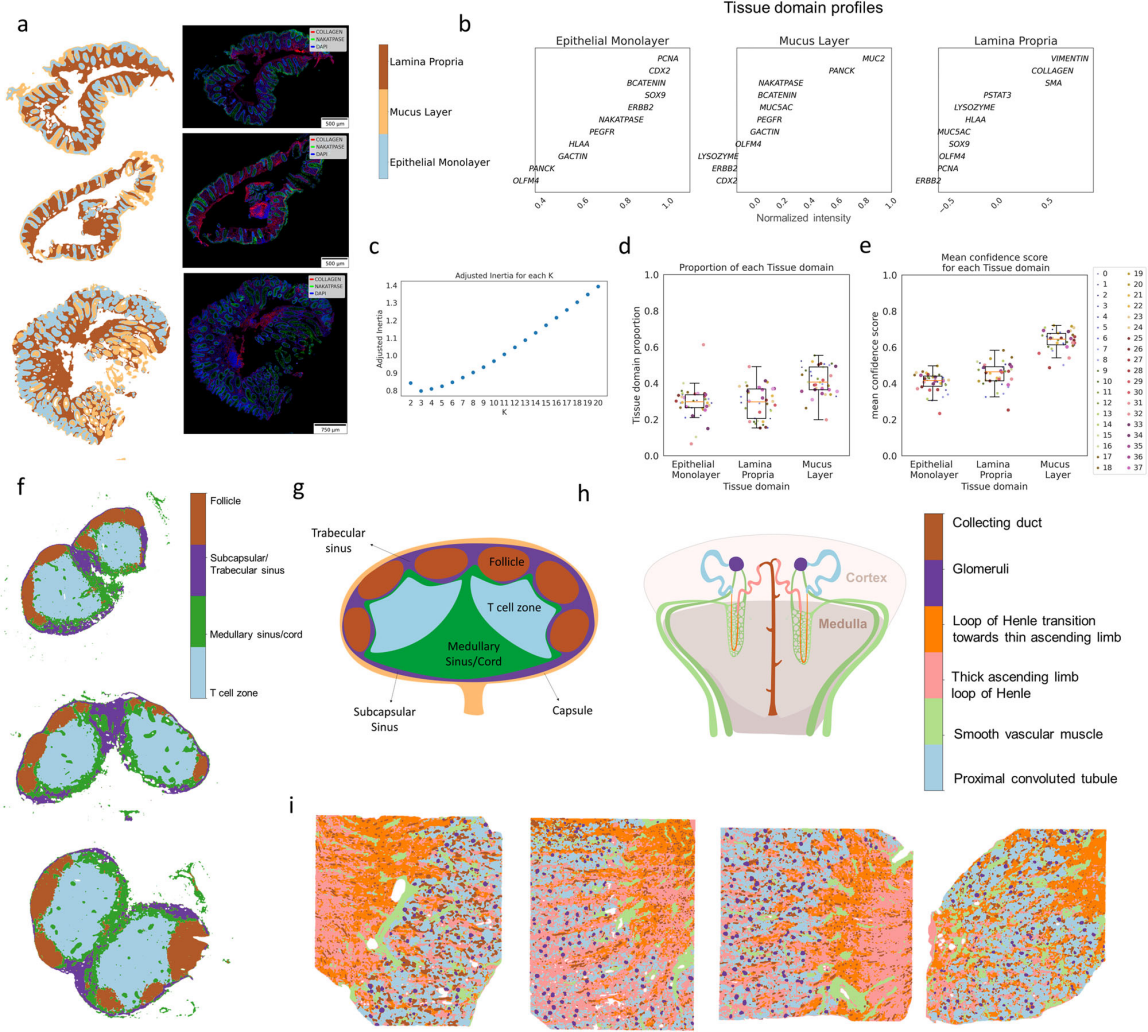
TD detection in multiple mIF datasets from various tissues and platforms. **a** Three representative colon mIF images with labeled tissue domains (α = 0.05) (left) and mIF images overlaid with Collagen, Na^+^–K^+^ ATPase, and DAPI (right). **b** Domain profile describing marker composition of each TD. **c** Estimated number of tissue domains in adjusted inertia plot. **d** Boxplot for proportion of each TD across 38 samples with each colored dot corresponding to a sample (legend on the right) (**e**) Boxplot for average confidence score across all pixels in each image for each TD with each color dot corresponding to a sample (legend on the right). **f** Lymph node CODEX images with labeled tissue domains (α = 0.05). **g** Illustration of lymph node depicting TD organization and (**h**) illustration of kidney depicting TD organization. **i** Human kidney CODEX images with labeled tissue domains (α = 0.05).

We applied MILWRM to mIF data generated for the Human Tumor Atlas Network (HTAN) consisting of human normal colon and different colonic pre-cancer subtypes (conventional adenomas—AD and serrated polyps—SER)^30^. These data comprised multichannel fluorescent images from 37 biospecimens consisting of tissues with different morphologies and pathological classifications confirmed by two pathologists (Supplementary Table 1). We performed a low-resolution application of MILWRM using a smoothing parameter (sigma) of 2 after downsampling the images by a factor of 16 to an isotropic resolution of 5.6 µm/pixel and with a penalty parameter of 0.05 (see “Materials” and “Methods”) that resulted in three tissue domains according to adjusted inertia, as illustrated by three representative samples (Figs. 2a–c and S1). According to domain profiles (Fig. 2c), the epithelial monolayer compartment was identified by markers such as CDX2, β-catenin, Na^+^–K^+^ ATPase, and proliferative marker PCNA, consistent with a high turnover hind-gut epithelium^31,32^. The mucus layer was enriched in MUC2, a secreted mucin^33–35^. The lamina propria region, where stromal cells are prominent, was identified by vimentin and collagen^36^. The results from MILWRM analysis are consistent with the tissue architecture of the colonic mucosa, as well as other mucosal tissues in the body.

MILWRM consistently identified these regions across the 37 tissue samples (Fig. 2d). To assess the quality of TD identification, MILWRM calculates a modified silhouette-based confidence score per pixel, which evaluates the deviation of each pixel from the centroid of the matched TD relative to the next closest *k-*means centroid. Most pixels across all samples have high confidence scores apart from a few in the epithelial and mucus tissue domains (Figs. 2e and  S2a). Low confidence scores can be attributed to inherent biological heterogeneity within epithelial domains, as the analysis is performed over samples from mixed pathological categories (normal, AD, and SER). Additionally, pixel-level data across samples intermixed in UMAP-embedded space illustrating removal of batch effects between images (Fig. S2b), thus demonstrating the ability of MILWRM to identify consensus regions over multiple samples. MILWRM captured about 80% of the variance in the multidimensional imaging data without any notable outliers, indicating that information within the imaging data is retained after MILWRM analysis (Fig. S2c). Thus, MILWRM performed on a cohort of 37 biospecimens was able to provide physiologically relevant tissue domains with high confidence.

Next, we obtained new CODEX imaging data from mouse lymph nodes (*N* = 3) and then applied MILWRM analysis. Lymph nodes are secondary lymphoid organs with well-defined substructures that support adaptive immunity. Broadly, these substructures consist of the cortex densely packed with lymphocytes and the medulla where vasculature and lymphatic sinuses are located^37^. We downsampled these images by a factor of 8 to an isotropic resolution of 2.6 µm/pixel and applied MILWRM (sigma = 2 and alpha 0.05, see “Methods”) which resulted in the detection of four tissue domains that described different areas of a lymph node (Figs. 2f and S3a). The lymph node cortex is composed of follicles containing densely packaged B cells and follicular dendritic cells that are marked by expression of B220 and CD21-35 protein, respectively^38,39^, a deeper paracortex enriched with CD3^+^ T cells and CD11c^+^ dendritic cells, a subcapsular/trabecular sinus zone enriched with CD169^+^ macrophages^40^, and a medullary region marked by the expression of stromal Collagen and CD31, marker for endothelial cells^41^ (Fig. S2d). These observations corroborate the general structure of the lymph node (Fig. 2g)^42^.

We also collected additional CODEX imaging data from human kidneys (*N* = 4)^43^ as part of the Human BioMolecular Atlas Program^44,45^ to test the MILWRM application (Fig. S3b). The kidney, an organ that is a central component of the urinary system, comprises more than a million functioning nephron units, each possessing diverse substructures that span the medulla and cortex regions of the kidney^46^. MILWRM detected six tissue domains in downsampled kidney images (resolution 10.2 µm/pixel) at a parameter setting of sigma = 2 and alpha = 0.05 (see “Methods”). As expected, MILWRM detected tissue domains that corresponded to different substructures of the nephron such as tissue domains of glomeruli marked by the expression of synaptopodin and proximal tubules marked by the expression of aquaporin 1 and CD90 (Figs. 2h–i and S2e)^43,47^. Similar to tissue domains in the lymph node, the tissue domains detected by MILWRM effectively capture the organization of both the medulla and cortex regions of the kidney, along with the intricate substructures contained within. MILWRM also detected two distinct regions of the loop of Henle—thick ascending limb marked by the expression of Uromodulin protein and the transition region towards the thin ascending limb—highlighting MILWRM’s ability to detect intricate tissue domains in the kidney (Fig. 2h–i). Collectively, these results demonstrate MILWRM’s versatility in detecting consensus tissue domains from a variety of tissues and organs using datasets collected from different imaging platforms.

### MILWRM identifies tissue domains associated with colon precancer subtypes

**Fig. 3:**
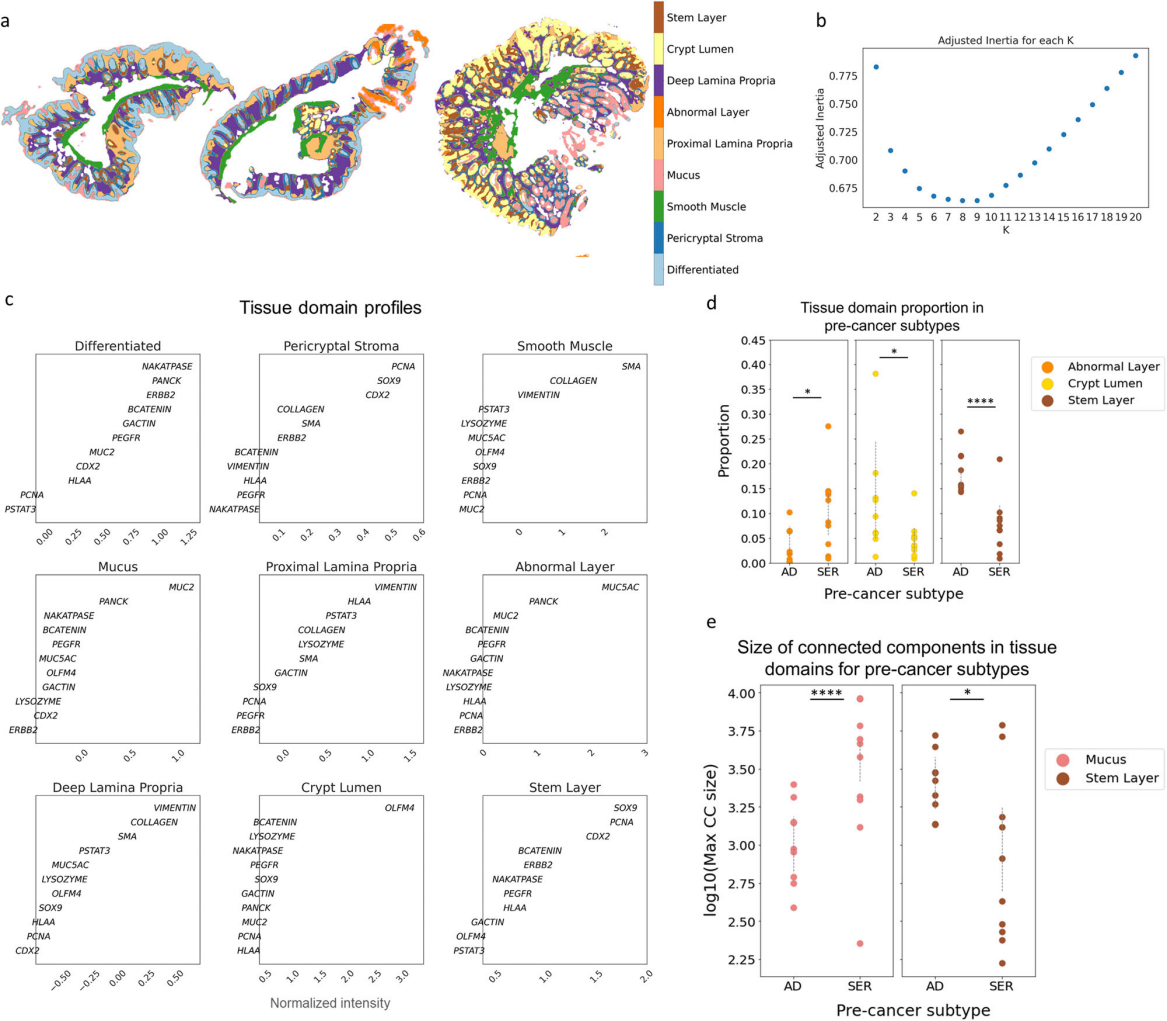
MILWRM tissue domains describe the molecular distinction between human colon adenoma pre-cancer subtypes. **a** Three representative colon mIF images with labeled tissue domains (α = 0.02). **b** Estimated number of tissue domains in adjusted inertia plot. **c** Domain profile describing marker composition of each TD. **d** Scatter plot for proportion for tissue domains that are significantly associated with pre-cancer subtypes. **e** Scatter plot for the log of size of maximum connected components for tissue domains that are significantly associated with pre-cancer subtypes. *Signifies a *p* value < 0.05 and ****signifies a *p* value < 0.0001.

In order to obtain more refined tissue domains that appropriately stratify the heterogeneous pathological categories of our colon samples (normal, AD, and SER), we next performed MILWRM with a reduced penalty parameter to increase the cluster resolution and model associations with polyp type (penalty parameter = 0.02, see “Methods”). We obtained nine MILWRM tissue domains that further broke down the epithelial compartment into stem (SOX9, PCNA, and CDX2), differentiated (Na^+^–K^+^ ATPase, PANCK, and β-catenin), mucus (MUC2), abnormal (MUC5AC+/PANCK+), and crypt lumen (OLFM4+), and the non-epithelial compartment into smooth muscle, pericryptal stroma, and proximal and deep lamina propria (Figs. 3a–c and S4). Interestingly, pericryptal stroma was identified with a mixture of epithelial and stromal markers and labeled a thin fibroblast layer comprising telocytes constituting the stem cell niche (Figs. 3a–c and S5a–e)^48,49^. Notably, the MILWRM epithelial tissue domains aligned with cell states, and their markers were identified in the same tissues using single-cell RNA-sequencing in our previous HTAN study^30^.

Using tissue classification consensus by two pathologists as ground truth, we then asked whether the two pre-cancer subtypes, AD and SER, have any differences in the organization of MILWRM tissue domains. We used generalized estimating equations (GEE) to statistically model the association of MILWRM TD proportions with tumor type and found a significant association between MILWRM proportions for crypt lumen, abnormal, and stem classes (Supplementary Table 2 and Fig. 3d). Specifically, ADs were associated with higher proportions of pixels labeled as stem and crypt lumen classes, consistent with their characteristic increased stemness driven by WNT-signaling^30,50^. In contrast, serrated polyps were associated with increased pixel proportions of the abnormal tissue class marked by MUC5AC; MUC5AC is a foregut endoderm mucin characteristic of metaplasia associated with serrated polyps^51^.

AD arises from stem cell expansion; these cells inevitably fill the entirety of abnormal crypts^30^. Therefore, we hypothesized that the stem MILWRM domain would display notably enhanced pixel connectivity because the absence of differentiated cell domains in AD would result in uninterrupted stem cell domains. We again used GEE to estimate the population average effect of pre-cancer subtype on MILWRM the maximum size of tissue-connected components (Supplementary Table 3 and Fig. 3e) and found a significant association between the size of connected components of stem and mucus tissue domains and pre-cancer type. The stem domain was expectedly more connected in the AD subtype whereas higher connectedness in the mucus domain was associated with the SER pre-cancer type. ADs have defects in the differentiation of goblet cells that inherently deplete the mucus layer^52–57^. This aligns with the association of AD with decreased connected mucus components as MUC2 expression decreases in adenoma during progression, which disrupts the normally contiguous mucus barrier in normal colonic and SER mucosa^58^ (Fig. 3e). No such association was observed for the connectivity of the abnormal MUC5AC+ domain since it comprises sporadic abnormal cells associated with secretion. These results align with recent atlas results demonstrating that ADs arose from stem cell expansion and serrated polyps from pyloric metaplasia^30,51^. The association of ADs with stem domains, both in prevalence and connectivity, is consistent with mouse models where labeled stem cells are observed to significantly expand in premalignant tumors driven by hyperactive WNT signaling^30,36^.

### MILWRM applied to ST reliably identifies tissue domains across multiple mouse brain cross-sections

**Fig. 4:**
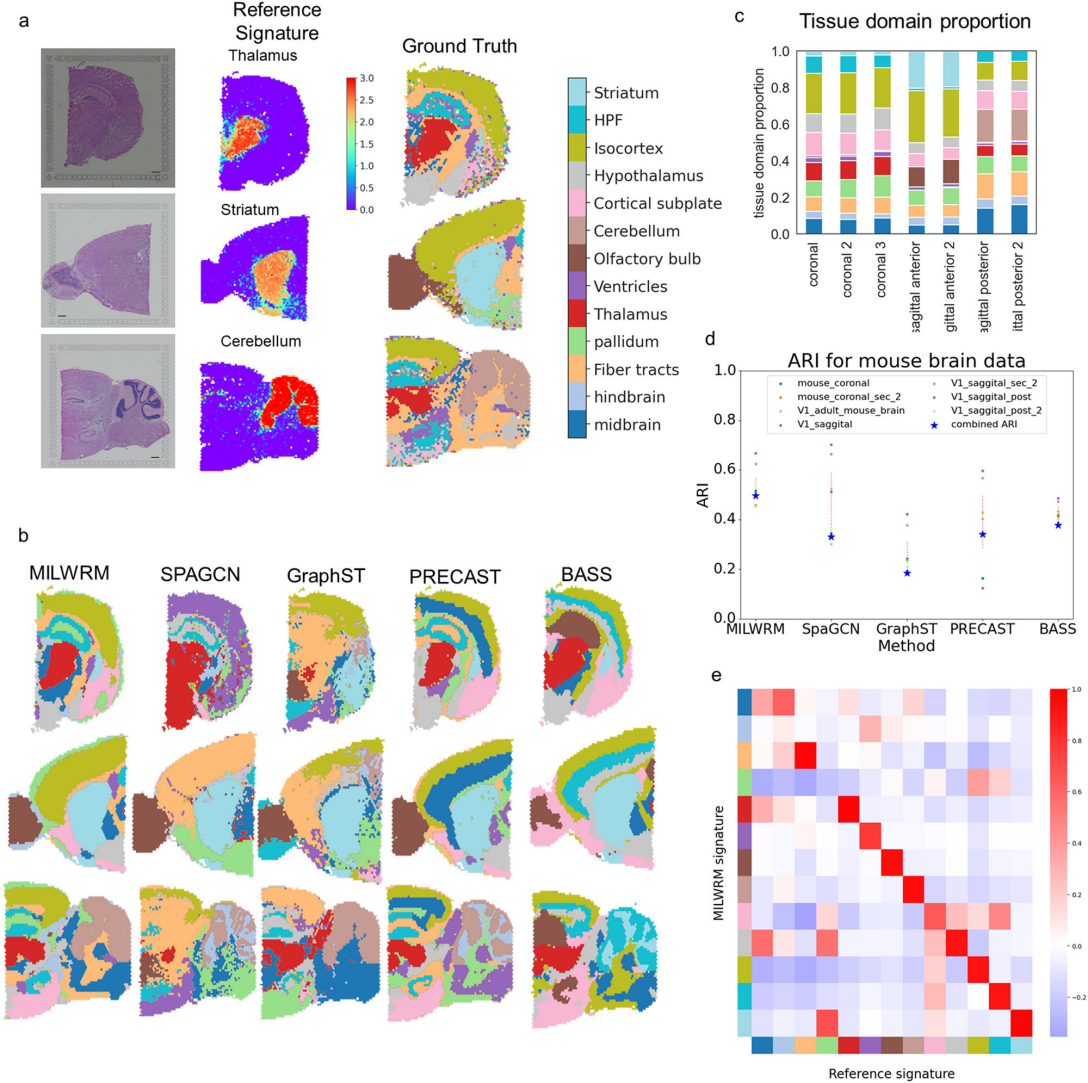
MILWRM detects consensus tissue domains in ST data from different mouse brain cross-sections. **a** Reference signature (middle—thalamus, striatum, and cerebellum top to bottom, respectively) based ground truth annotation (right) in mouse brain ST data (scale bar = 500 µm). **b** MILWRM, SpaGCN, GraphST, PRECAST, and BASS detected tissue domains (*k* = 13) overlaid on three mouse brain ST samples. **c** Proportion of tissue domains in slides each bar corresponding to the legend on the left. **d** Scatter plot for ARI and consensus ARI. **e** Correlation matrix for overall correlation between MILWRM and reference scores for each TD and anatomical region across all spots.

To benchmark MILWRM against state-of-the-art TD identification methods (SpaGCN, GraphST, PRECAST, and BASS), we utilized a 10× Visium ST dataset generated from the mouse brain. For assessing the performance of these methods, we first created a ground truth for each brain histological region by curating a reference gene list from differential gene expression data acquired using in situ hybridization by the Allen Brain Atlas^59^. For histological regions not available in the Allen Brain Atlas, we curated reference gene lists from another source, the Molecular Atlas of the mouse brain^60^. To validate these reference gene lists, we computed a signature score for the curated gene list for each brain region, and then overlaid these signatures onto ST data (Fig. 4a)*.* As expected, the reference gene signatures were highly specific to respective histological regions. We used these scores to assign histological region labels to each spot in this dataset which serves as ground truth for this dataset (Fig. 4a and see “Methods”).

We applied MILWRM and other methods to all the samples simultaneously using *k* = 13 based on ground truth annotations (see “Methods”). MILWRM identified consensus tissue domains across samples that overlap with ground truth annotations closely (Fig. 4b). Notably, MILWRM identified consensus domains despite differences in the orientations and cuts of brain tissue sections. For example, MILWRM was able to capture tissue domains that are unique only to certain sections, such as the cerebellum specific to sagittal-posterior cut, as well as domains with diverse shapes and sizes due to orientation differences, such as the striatum that is small in the coronal slice, large in the sagittal-anterior section, and absent in the sagittal-posterior section (Figs. 4c and S6a). Compared to MILWRM, SpaGCN, and GraphST either fail to identify consensus tissue domains across different sections (i.e., isocortex in the case of SpaGCN) or do not recognize certain regions at all, such as missing the thalamus for both methods. On the other hand, PRECAST and BASS identified intricate brain regions at a higher resolution than the ground truth. However, both methods also missed a number of difficult domains such as ventricles. Interestingly if we increase the clustering resolution by reducing the penalty parameter for MILWRM, it is also capable of identifying finer brain structures while not missing more difficult regions such as ventricles (Fig. S6b).

We also compared the performance of each method using the adjusted rand index (ARI) that measures the similarity between two clustering results. We first computed ARI for each method against the ground truth per sample. Additionally, to quantitatively compare if the labels were consistent across samples, we computed a consensus ARI by calculating the ARI for all the labels collectively. MILWRM has a higher ARI than the rest of the methods across most samples (Fig. 4d) and the highest consensus ARI (Fig. 4d, blue star). We also compared the signatures identified by MILWRM to the reference gene signatures used for ground truth annotations. To quantify the performance between reference gene signatures and MILWRM signatures, we calculated a spot-by-spot correlation of the two sets of signature scores across all slides. The high correlation between the MILWRM and reference scores was observed on a brain region-specific basis (Fig. 4e). These results illustrate that the MILWRM approach can be effectively applied to genome-scale ST data for extracting TD-specific molecular information.

### MILWRM applied to annotated DLPFC ST data performs better than the state-of-the-art methods

**Fig. 5:**
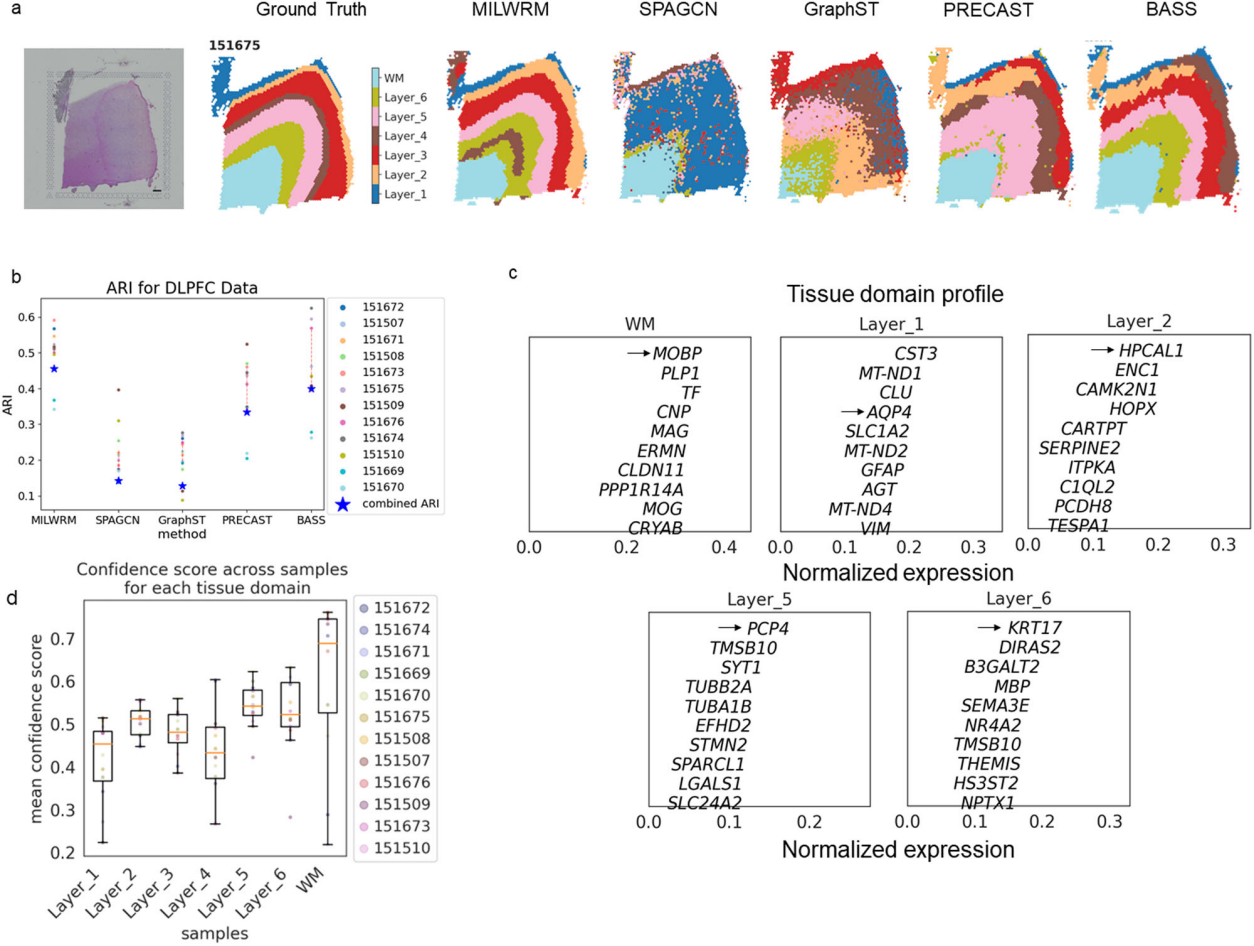
MILWRM applied to annotated DLPFC ST data performs better than the state-of-the-art methods. **a** DLPFC sample 151675 (scale bar = 500 µm) with ground truth, MILWRM, SpaGCN, GraphST, PRECAST, and BASS results overlaid (*k* *=* *7)*. **b** Scatter plot for ARI and consensus ARI. **c** TD profile describing the genes enriched in each TD. **d** Boxplot for average confidence score across all pixels in each image for each TD with each color dot corresponding to a sample (legend on the right).

The ground truth created for the mouse brain dataset was based on the expression of region-specific genes collected from other well-curated sources without accounting for the histomorphology of tissue substructures. Thus, we wanted to further benchmark MILWRM against other methods (SpaGCN, GraphST, PRECAST, and BASS) in a dorsolateral prefrontal cortex (DLPFC) dataset that was manually annotated based on both region-specific gene expression and the morphology of different DLPFC layers^61^. We applied MILWRM and the rest of the methods collectively to all the samples together with a fixed *k* = 7 based on ground truth annotations. MILWRM more accurately identified all seven key layers in the given sample compared to SpaGCN, GraphST, and PRECAST, which either did not identify layers of DLPFC properly (SpaGCN and GraphST) or identified uneven layers (PRECAST) (Fig. 5a). BASS performed better than SpaGCN, GraphST, and PRECAST and had comparable performance to MILWRM (Fig. 5b). However, MILWRM had better overall performance based on the consensus ARI and had more consistent performance across slides than BASS (Fig. 5b). Although MILWRM did well comparatively, there were still some discrepancies between its results compared to manual annotation. Layers unique to section 151675, such as layer 1, were misidentified in the other sections (Figs. S7–S9), and layer 4 was not captured accurately. This error likely occurred because that layer represented a relatively small component of the total variance and was missed by the *k*-means plus smoothing algorithm. Interestingly, the TD profile for specific layers presented layer-enriched differentially expressed genes discovered by Maynard et al., for different layers in the DLPFC dataset (Fig. 5, black arrow). Overall, these results demonstrate that MILWRM performs better than current state-of-the-art methods for TD detection in ST data.

## Discussion

Pixel-based TD detection forms the basis of the top–down approach to spatial data analysis. Current methods of TD detection are either based on a bottom-up approach, that is, building cellular neighborhoods using segmented single-cell data^11,12,62^ and/or lack scalability across samples^23–25,63,64^. Here, we addressed this gap by developing MILWRM, an algorithm to detect spatial domains across samples through a top–down, pixel-based approach. We demonstrated the applicability of MILWRM to find relevant biological phenotypes in multiple data modalities (mIF and ST) in an unsupervised way without manual thresholding and annotation.

MILWRM application in kidney and lymph nodes resulted in tissue domains that captured the biological organization of the tissue i.e., the presence of a large T cell zone in the center of lymph nodes right above the medullary regions and glomeruli only present in the cortex region of the kidney alongside proximal tubules. Additionally, MILWRM identified tissue domains of varying sizes in these datasets such as glomeruli in the kidney data.

While abnormal tissues can be distinguished from normal tissues within a slide using other methods, MILWRM’s identification of consensus domains across slides has significant value. When there are specimens that are completely composed of abnormal tissues, comparison between specimens (normal vs abnormal) is the only way to distinguish between disease states. We demonstrated that MILWRM is able to discern organizational differences in tissue domains related to disease subtypes. Finally, MILWRM was able to identify consensus domains and gene lists that match with organ anatomical features despite different cuts and orientations. This is important because the tissue structure from individual cuts may appear morphologically different when it is functionally identical. These examples showed the real-world application of MILWRM in the pathological diagnosis of disease subtypes and anatomic classification and characterization.

mIF data present additional pixel analysis obstacles that make implementation of existing tools for ST data impossible. First, due to lower marker dimensionality, marker selection, and management is of utmost importance. Unlike ST data where vectors of genes define programs and phenotypes, mIF phenotypes are usually defined by single markers. Highly expressed markers may mask lower expression markers if suboptimal preprocessing is performed, thus preventing some tissue domains from being detected. Secondly, high-resolution microscopy data are generally incompatible with pixel-based algorithms built for low-resolution ST data, such as SpaGCN. The creation of image tiles or large-scale downsampling is needed to satisfy speed and memory requirements. In contrast to most state-of-the-art methods for pixel-based analysis that are data type-specific, we demonstrated that MILWRM is adaptable to multiple imaging data types and is scalable to many samples.

When applied across multiple ST samples, most of the existing domain detection methods failed to recognize meaningful tissue domains or could not reach consensus. Additionally, MILWRM also provides the ability to perform tissue clustering at different levels of smoothing, downsampling, and cluster resolution.

It is important to note the limitations of the MILWRM algorithm, which can help users identify when it will be effective in their data. First, although MILWRM outperformed other comparable methods of TD detection, there are still inaccuracies when compared to ground truth identified by expert annotation. When applied to the DLPFC dataset, MILWRM did not identify layer 4 in any specimen (Figs. 5a and S7–S9). This is supported by the various QC metrics that MILWRM provides, which show that the confidence scores are lowest in misidentified layers (Fig. 5d). This misclassification is likely due to MILWRM’s reliance on *k*-means and smoothing for proximity information, which lowers effective resolution and can potentially result in small domains being missed when they are heterogeneous in size. Using a different data representation that incorporates spatial information in a direct way without dimensionality reduction may be more useful in the case of very high dimensional data, such as Visium ST^60^. Second, *k*-means clustering can yield unusual outcomes if the marker space structure is non-Euclidian^65^. In the event of poor performance, MILWRM’s quality control metrics may help to recognize such cases. Finally, as MILWRM is solely based on gene expression, it does not incorporate other features, such as histomorphology, for TD identification.

The emergence of spatially resolved assays has offered unique insights into tissue organization, with cohort studies in a medium throughput manner being a possibility. MILWRM is a tool tailored for multi-sample investigations, that excels in reducing biases caused by a reliance on single-cell segmentation, in a user-friendly, easily interpretable manner across diverse imaging modalities. Despite its limitations, MILWRM outperformed other methods in identifying consensus tissue domains across samples and is a fast and versatile package that is widely applicable to many modalities of spatial profiling. The results demonstrate that MILWRM provides interpretable tissue domains across heterogeneous samples and that the domains are sensitive to biological differences between samples and tissue types within samples.

## Methods

### MILWRM—data preprocessing

Spatial-omics data differ in their acquisition and technological artifacts across modalities, so these preprocessing steps are data type specific. It is important for the user to understand and apply methods that are reasonable for their modality before using MILWRM, otherwise, results can be corrupted by batch effects.

### MILWRM—data preprocessing for mIF

Prior to preprocessing, mIF data were scaled from uint8 (0–255) to float (0–1) and downsampled by a factor of 1/16th for the human colon, 1/8th for lymph node and 1/32th for Kidney dataset, resolution to speed computation and normalization process. There is no sacrifice in the quality of the neighborhood identification by downsampling as mIF data have subcellular spatial resolution and MILWRM is designed to identify broad tissue domains. After downsampling we created tissue masks for each image as described in mIF tissue mask generation with MILWRM. Finally, we applied image normalization at the slide level using the formula $y\,=\,{\log }_{10}\left(\frac{x}{{\mu }_{x}}\,+\,1\right)$, where *x* is the unnormalized data and ${\mu }_{x}$ is the mean of non-zero pixels in the image, per marker. This normalization was a modification of an existing method evaluated in segmented mIF data. Here, we implemented the mean of non-zero pixels to accommodate channels with sparse signal intensities^66^. The downsampling performed on images prior to this normalization step also aligns with the unbiased grid-based normalization framework described by Graf et al. ^67^. To incorporate spatial information within each pixel, after normalization, we applied Gaussian smoothing. The radius of blurring can be controlled by adjusting the sigma parameter in MILWRM for mIF modality. Here, we use sigma = 2 for smoothing.

### MILWRM—data preprocessing for ST

The above-described steps differ slightly between the mIF and ST modality. For ST data the first step is to reduce the dimensionality of the transcriptomics data. For the analysis shown in this paper, we used principal component analysis (PCA) for dimensional reduction, but other methods can also be used with MILWRM, such as non-negative matrix factorization^68^. The number of PCs was selected visually using the variance ratio rank plot (12 and 10 for the mouse brain and DLPFC, respectively). We used Harmony^69^ to correct technical variations between the samples. As in mIF, blurring is applied to the ST slides to preserve spatial information. To perform blurring, each central spot is assigned the average value for the selected reduced components (PCs in this case) across the spots within the neighborhood of the central spot. The spatial neighborhoods are computed using the Squidpy Python package^70^. The neighborhood distance can be controlled by adjusting the n_rings parameter. Here, we use n_rings = 1 for smoothing in ST data.

### MILWRM—identification of tissue domains

The tissue domains in the data are identified across slides by performing *k*-means clustering on the preprocessed data. MILWRM reduced computation time by randomly subsampling pixels within the tissue mask for mIF modality. Pixels are sampled uniformly from within the pixel mask at a default proportion of 0.2 per sample without regard for spatial location. The fraction of pixels or all the spots are serialized to build the *k*-means model. If dimension reduction is performed, then the input data are the PCA components, otherwise, the input is the batch-adjusted marker channels. After downsampling and PCA, prior to performing *k*-means, the data are *Z*-normalized to ensure that the mean and variances are similar across the different channels/PCA components of the input data. The *k*-selection for *k*-means is done by estimating the adjusted inertia metric. Adjusted inertia is inertia weighted by a penalty parameter that controls the number of clusters^29^. For MILWRM, the parameter can be adjusted to control the resolution of tissue domains identified.

After performing *k*-means clustering, tissue domains are identified in the full dataset by assigning the TD for the closest cluster centroid from the *k*-means model. The mean and variance computed for subsampled data are used to *Z*-normalize the original image data. By performing *k*-means model estimation in the subsample, MILWRM can reduce computational demand for mIF modality. *k-*Means is performed on the entire dataset in the ST modality.

### MILWRM—quality control and tissue labeling

Once the regions are identified it is useful to label the tissue domains based on their marker expression profile and assess the quality of clustering. The cluster centroids for each TD are plotted in marker or PCA component space to label the tissue cluster based on its expression profile. The centroids can also be plotted in gene space for ST modality or other dimensionally reduced components. The quality of clustering is assessed at the whole slide and pixel levels. To assess the whole slide fit, we compute the variance explained and the mean square error within each slide. These metrics allow the user to flag slides for manual review where the overall fit might be bad. We also compute pixel-level confidence score using the formula $y\,=\,\frac{{{{{{{\rm{dist}}}}}}}_{x,{c}_{2}}\,-\,{{{{{{\rm{dist}}}}}}}_{x,c}}{{{{{{{\rm{dist}}}}}}}_{x,{c}_{2}}}$ where ${{{{{\rm{dist}}}}}}$ is the Euclidean distance between pixel or spot, $x$, assigned centroid, $c$, and the second closest centroid, $\,{c}_{2}$. The confidence scores take values between zero and one where higher values indicate a smaller distance between the assigned centroid and closest centroid thus, better fit. This metric is a fast simplification of the Silhouette index^71^.

### MILWRM—mIF tissue mask generation

MILWRM has a designated function to perform the creation of tissue masks through the MILWRM pipeline described above. Each preprocessing step is performed on individual images including log normalization and smoothening with a Gaussian filter (sigma = 2). Finally, the mask is created using Kmeans clustering with *n* = 2. The *k-*means cluster centers are then *Z*-normalized, and the cluster center with a mean smaller or equal to zero is set as background.

### Human colon mIF data acquisition

The mIF data were generated for the HTAN consisting of human normal colon and different colonic pre-cancer subtypes (conventional adenomas—AD and serrated polyps—SER)^30^. These data comprised multichannel fluorescent images from 37 biospecimens consisting of tissues with different morphologies and pathological classifications, as confirmed by two pathologists (Supplementary Table 1). Cyclical immunofluorescence staining, detection, and dye inactivation were performed as described previously^2^. In brief, fluorescent images were acquired at 200× magnification on a GE In Cell Analyzer 2500 using the Cell DIVE ® platform. Exposure times were determined for each antibody. Dye inactivation was accomplished with an alkaline peroxide solution, and background images were collected after each round of staining to ensure fluorophore inactivation. Staining sequence, conditions, and exposure times are as described in^30^. Following the acquisition, images were processed as described^31^. Briefly, DAPI images for each round were registered to a common baseline, and autofluorescence in staining rounds was removed by subtracting the equalized background image of the previous round for each position.

### Lymph node CODEX data acquisition

Mouse lymph nodes were embedded in OCT (Sakura) and frozen in liquid nitrogen. The lymph nodes were sectioned into 7 µm cryosections on coverslips prepared according to the manufacturer’s protocol (Akoya Biosciences). Antibody staining and fluorescent reporter plate preparation were carried out following the manufacturer’s protocol for fresh frozen tissue (Akoya Biosciences), with one modification: just prior to antibody addition, cryosections were incubated in staining buffer for 20 min, then Fc blocked with anti-CD16/32 (clone 93, 0.005 µg/µL) for 20 min at room temperature. Samples were then rinsed with staining buffer and incubated with the antibody cocktail according to Akoya’s protocol. We conjugated antibodies listed as custom conjugates to oligonucleotide barcodes from Akoya Biosciences according to the manufacturer’s protocol. Tiled images of the lymph node were acquired with a Leica DMi8 microscope using CODEX CIM software, and images were preprocessed using the CODEX MAV default settings.

### Kidney CODEX data acquisition

Human kidney tissue was obtained during a full nephrectomy performed at Vanderbilt University Medical Center (VUMC). The VUMC Cooperative Human Tissue Network (CHTN) acquired and processed the tissue in compliance with CHTN standard protocols. Institutional IRB policies were in place for consent to collect remnant tissue from participants. A detailed protocol for tissue processing, embedding, and storage is available^72^.Tissues were sectioned at 10 um thickness and probed with an antibody panel directed at specific renal targets. The antibody panel consisted of carrier-free, primary antibodies from Abcam (Cambridge, MA) conjugated to barcodes from Akoya Biosciences (Menlo Park, CA). Antibodies, barcodes, and the reporter channel for each antibody in the panel were CD90 (barcode 22, reporter AF 750), aquaporin 1 (barcode 002, reporter Atto 550), beta-catenin (barcode 003, reporter Cy5), calbindin (barcode 004, reporter AF 750), synaptopodin (barcode 006, reporter Cy 5), aquaporin 2 (barcode 015, reporter Cy5), alpha smooth muscle actin (barcode 029, reporter Atto 550), vimentin (barcode 042, reporter Cy5), uromodulin (barcode 047, reporter Atto 550), and cytokeratin-7 (barcode 005, reporter Atto 550). Antibody conjugation, tissue staining, and CODEX multiplex immunofluorescence methods were performed as described in^43^. Image processing was done using the Zeiss Zen Blue software package and multiplex immunofluorescence cycle registration was performed using *wsireg*^73^.

### Statistics and reproducibility

In order to assess the sensitivity of MILWRM regions to biological differences between precancer subtypes we computed tissue proportions and connected component statistics for each TD within the tumor region of each image and used GEEs to model how these variables were associated with precancer subtypes. Connected components were estimated for each image in Python using the label function in scipy.ndimage.measurements module. For the tissue proportions, we modeled each tissue proportion separately using a binomial family model assuming that images from the same slide had an exchangeable correlation structure. We modeled the maximum connected component size in order to quantify how the size and connectedness of different tissue domains differed across precancer subtypes. In these analyses, we used log transformation in a Gaussian family model with a log transformation on the maximum connected component size and included a log of the total tissue volume as a covariate. In all models, we weighted each region by its total image size so that results were not affected by noisy estimates from smaller images. Statistical analyses were performed in R using the geepack package^74^. We plotted all results with unadjusted significant *p* values and reported adjusted *p* values using the Benjamini–Hochberg procedure and a robust effect size index^75,76^ (Supplementary Tables 2 and 3).

### TD signature scores for ST data

The manual annotation for tissue domains in ST data was verified by generating signature gene scores specific to each brain region. For this purpose, we extracted differentially expressed genes from the Allen brain atlas for all available brain regions and the molecular atlas of adult mouse brain^60^ for the fiber tract and ventricles. To extract differentially expressed genes from Allen’s brain atlas we performed a differential search using mouse brain regions as target structure and entire gray matter as the contrast structure.

MILWRM also identified a set of genes for each TD. We computed a score for both the reference signature set and the MILWRM gene set using SCANPY^77,78^. After computing the reference signature for each spot, Argmax was used for the final annotations.

### Comparison with other methods

To evaluate MILWRM’s performance at TD detection, we compared it to other recently developed domain detection methods for ST data. We used the default parameters and preprocessing steps suggested for each method in their respective papers and the same number of clusters as the ground truth for the DLPFC and mouse brain dataset. We deemed this to be a reasonable choice as these methods all presented analyses of the DLPFC dataset and SpaGCN analyzed the mouse brain data as well. Our implementation of these algorithms differs in that we applied it across all slides in the dataset. Finally, the output labels for each sample were used to compute the ARI. To compute consensus ARI, we concatenated output labels for all the samples together and estimated ARI against the concatenated ground truth. Notably, PRECAST caused some unexpected trimming of a few samples in both DLPFC and mouse brain data. These samples were excluded from ARI quantification and downstream analysis.

### Materials availability

This study did not generate any new unique reagents.

## Supplementary information

Supplementary Information

## Data Availability

Imaging data can be found on Zenodo (https://zenodo.org/records/10557593). The 10× Visium mouse brain dataset can be downloaded from the 10× Visium website. The DLPFC dataset can be found here (https://github.com/LieberInstitute/HumanPilot).

## Code availability

The package is available to be installed directly from the PyPi website (https://pypi.org/project/MILWRM/). The source code for the package and code for the figures can be found on github (https://github.com/Ken-Lau-Lab/MILWRM).
