# Supplementary material for: Consensus tissue domain detection in spatial omics data using multiplex image labeling with regional morphology (MILWRM)
Source: Commun Biol. 2024 Oct 30;7:1295. doi: 10.1038/s42003-024-06281-8 (PMC11525554; doi:10.1038/s42003-024-06281-8)
Supplement: Supplementary file 1 — Supplementary Information [file 42003_2024_6281_MOESM1_ESM.pdf]

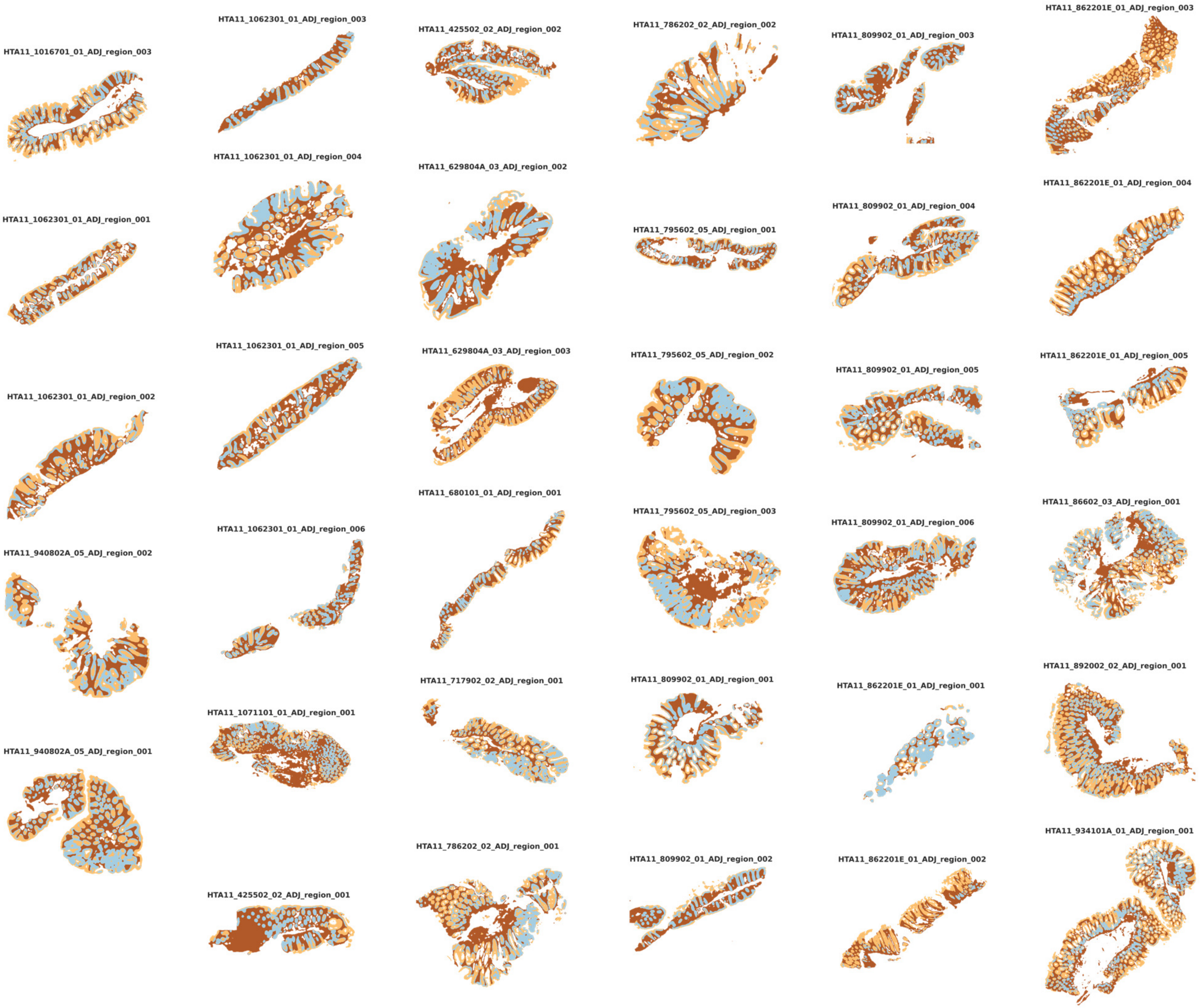

Figure S1. Tissue domain labels for human colonic samples (alpha = 0.05). Tissue domains for all the colonic samples (alpha = 0.05).

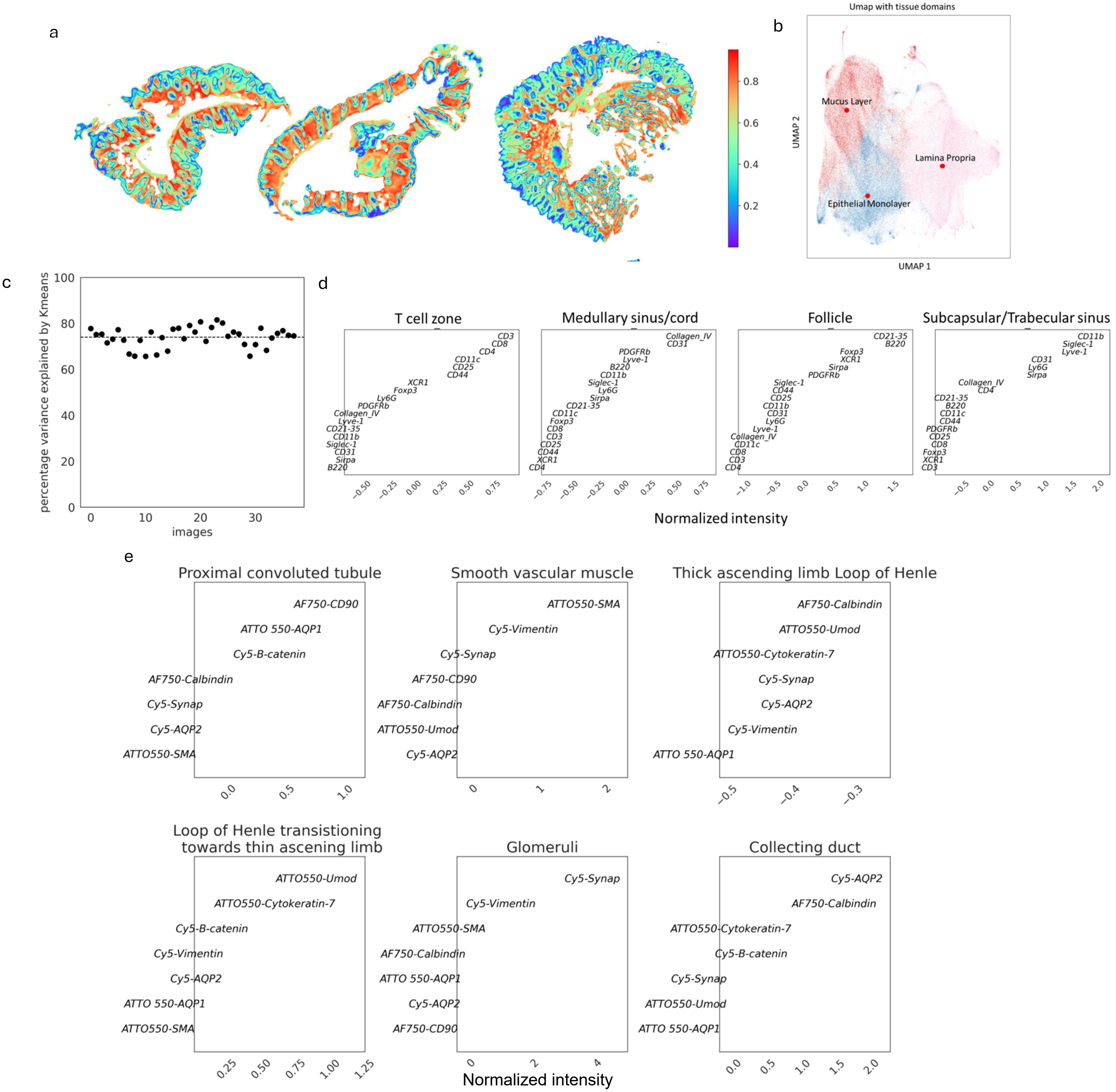

Figure S2. MILWRM QC metrics and domain profiles on colon, lymph node, and kidney. (a) Three representative colon mIF images with confidence score overlayed. (b) Umap of colon pixel data used for model building with tissue domain labels and centroids (different color overlays). (c) Scatter plot for percentage variance explained by the *k*-means model over 38 colon samples with each dot corresponding to a sample; dotted line is mean variance explained across all samples. (d) Domain profile describing marker composition of each tissue domain in the lymph node samples. (e) Domain profile describing marker composition of each tissue domain in the kidney samples.

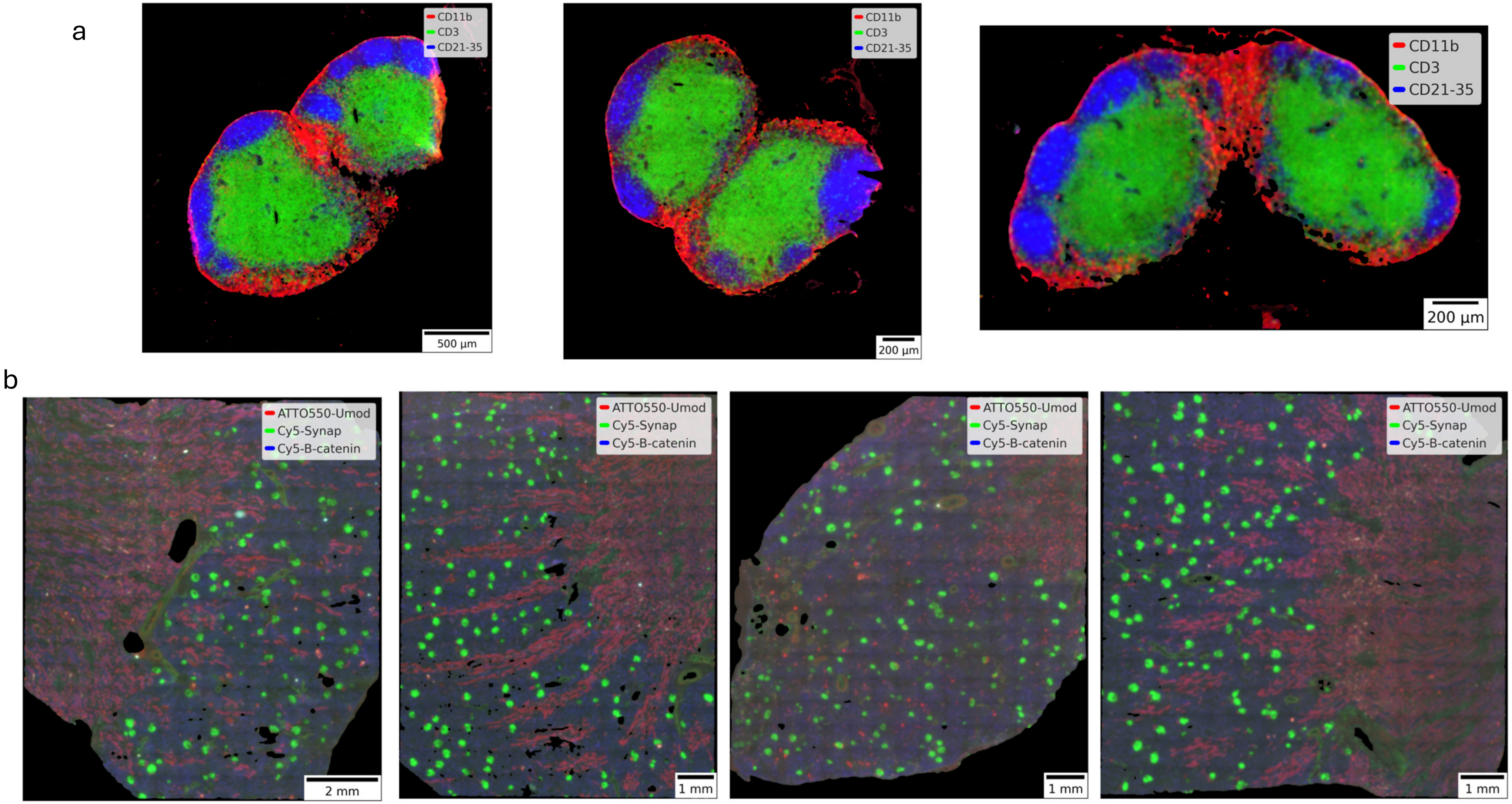

Figure S3. Kidney and lymph node CODEX images. (a) Lymph node CODEX images with CD11b, CD3 and CD21-35 overlayed. (b) Kidney CODEX images with Uromdoulin, Synaptopodin and β-catenin overlayed.

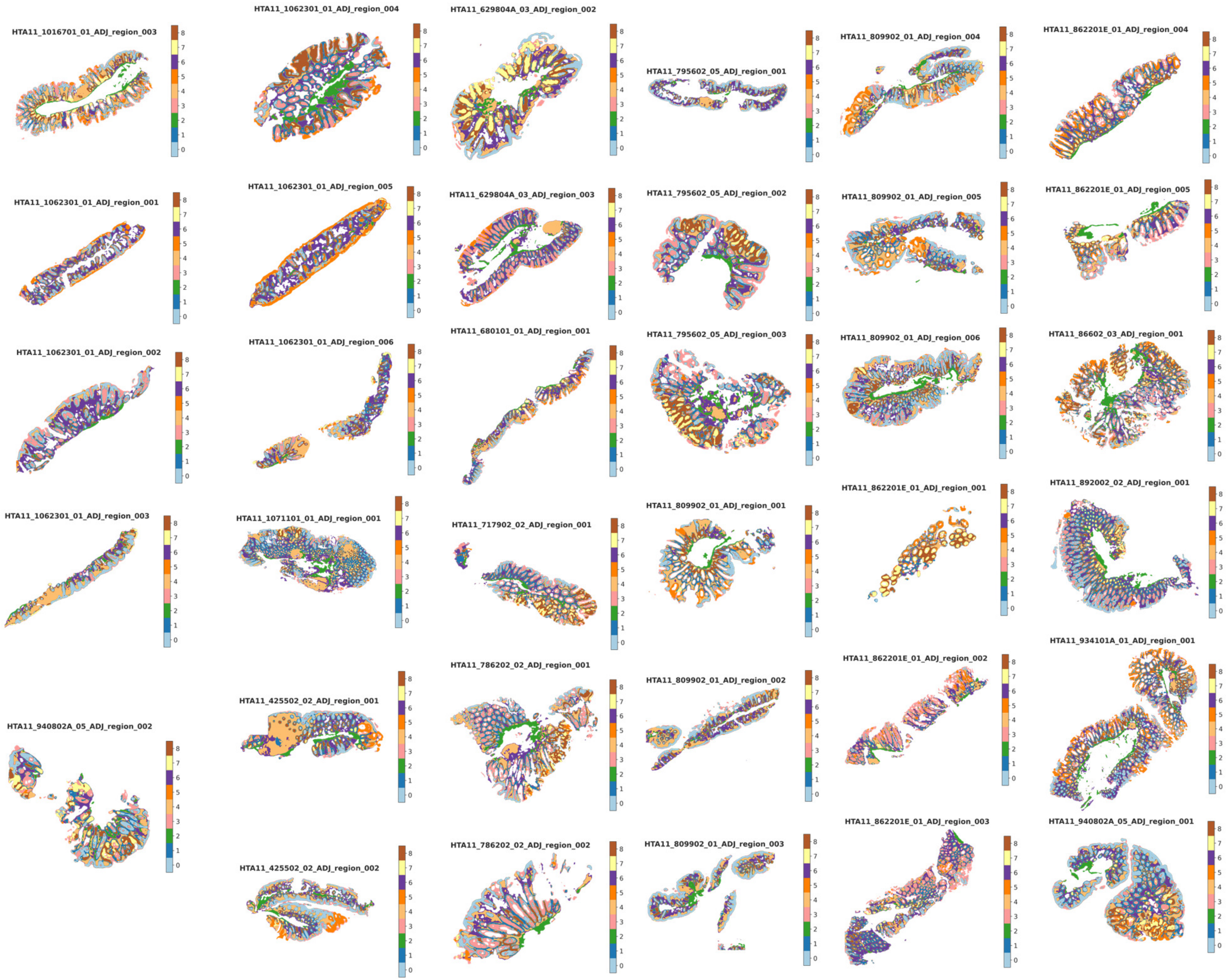

Figure S4. Tissue domain labels for human colonic samples (alpha = 0.02). Tissue domains for all the colonic samples (alpha = 0.02).

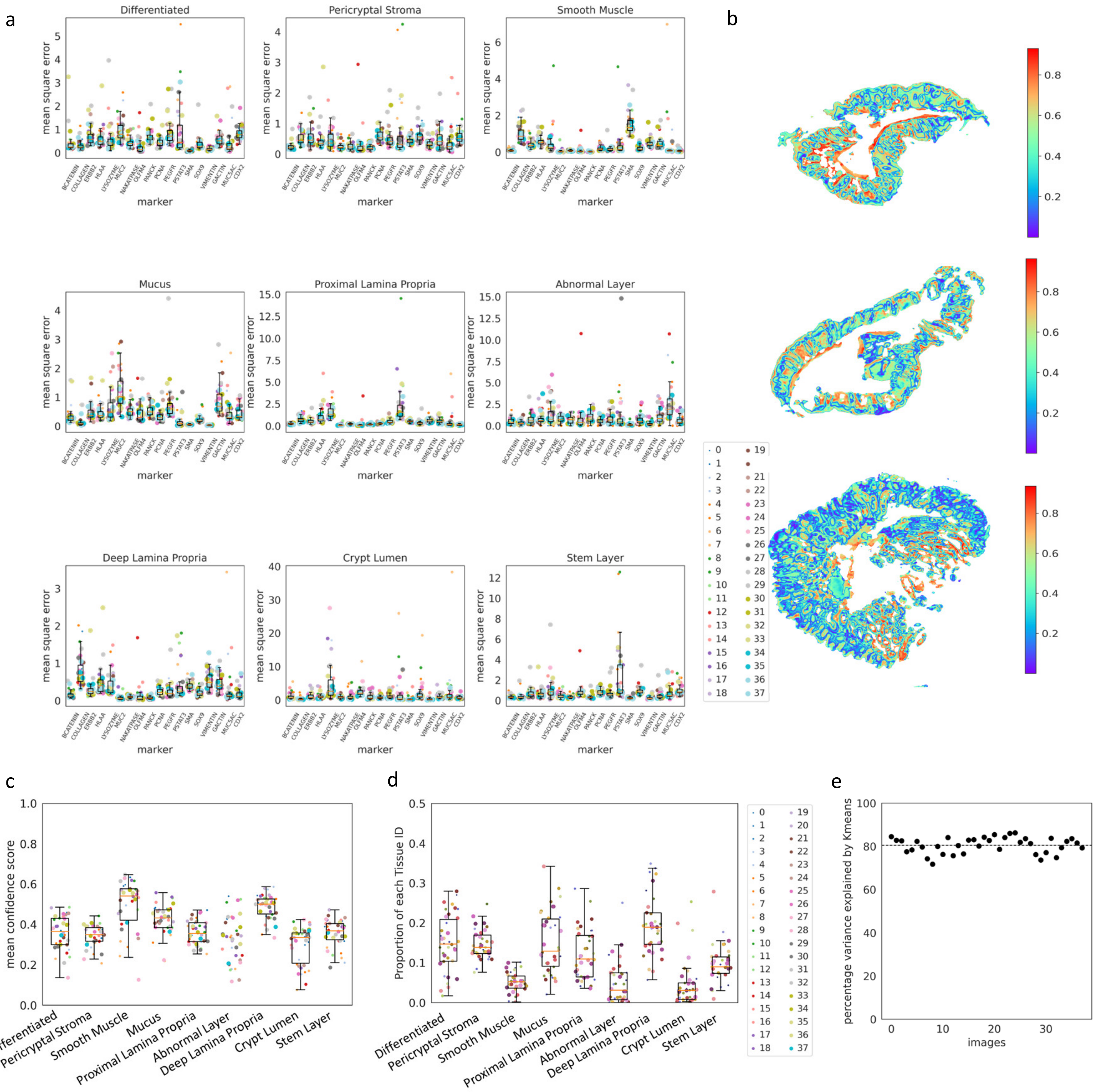

Figure S5. MILWRM QC metrics on colonic precancer specimens. (a) Boxplot for mean square error within each tissue domain for each marker. Mean square error is average of Euclidean distance between each pixel intensity of that marker and *k*-means centroid for that tissue domain. Colored dots represent a sample. (b) Confidence scores overlayed on three representative tissues. (c) Boxplot for average confidence score across all pixels in each image for each tissue domain with each color dot corresponding to a sample (legend on the right). (d) Boxplot for proportion of each tissue domain across 38 samples with each colored dot corresponding to a sample (legend on the right). (e) Scatter plot for percentage variance explained by the *k*-means model over 38 samples with each dot corresponding to a sample; dotted line is mean variance explained across all samples**.**

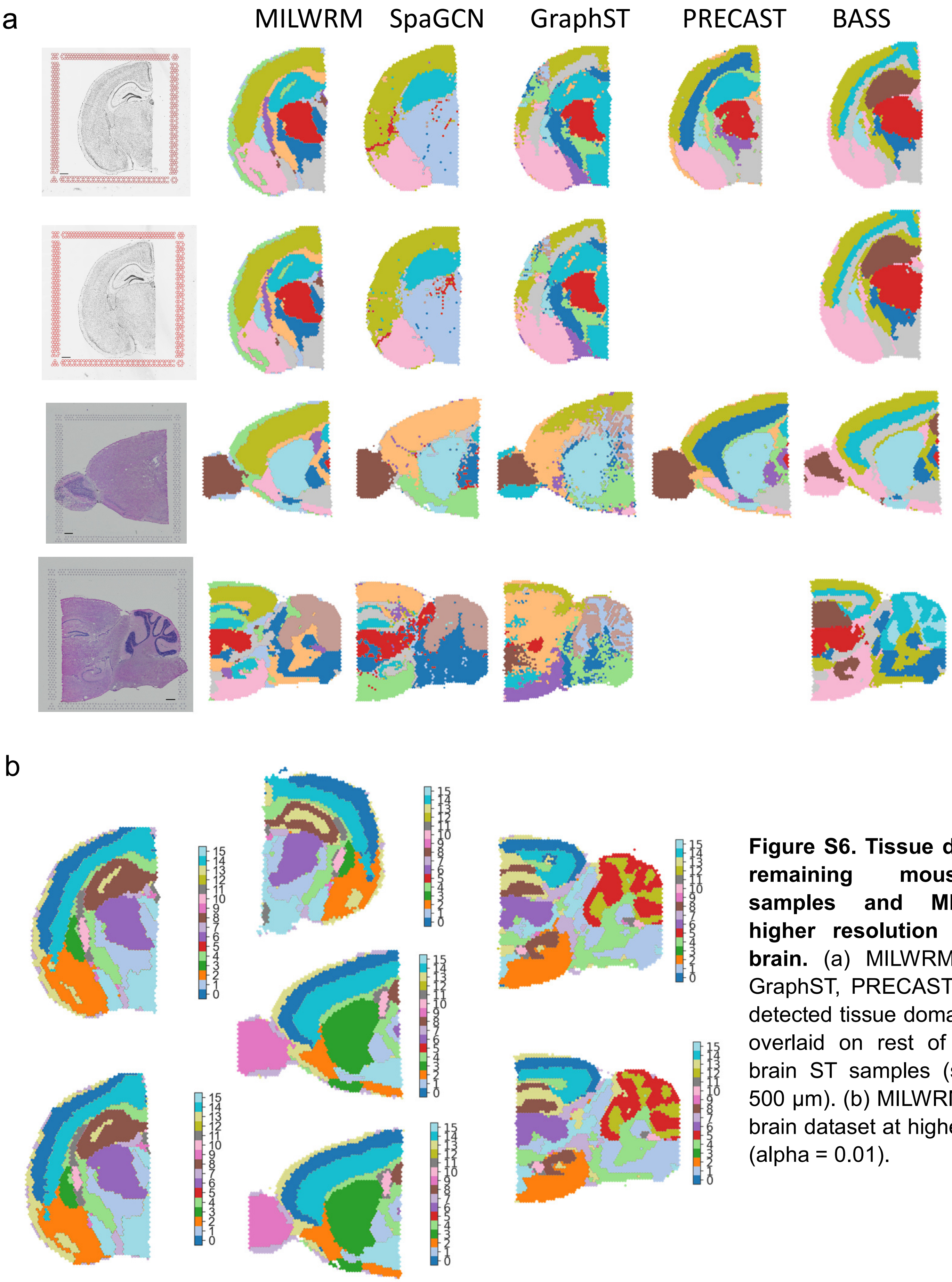

Figure S6. Tissue domains for remaining mouse brain samples and MILWRM at higher resolution on mouse brain. (a) MILWRM, SpaGCN, GraphST, PRECAST and BASS detected tissue domains (k = 13) overlaid on rest of the mouse brain ST samples (scale bar = 500 µm). (b) MILWRM on mouse brain dataset at higher resolution (alpha = 0.01).

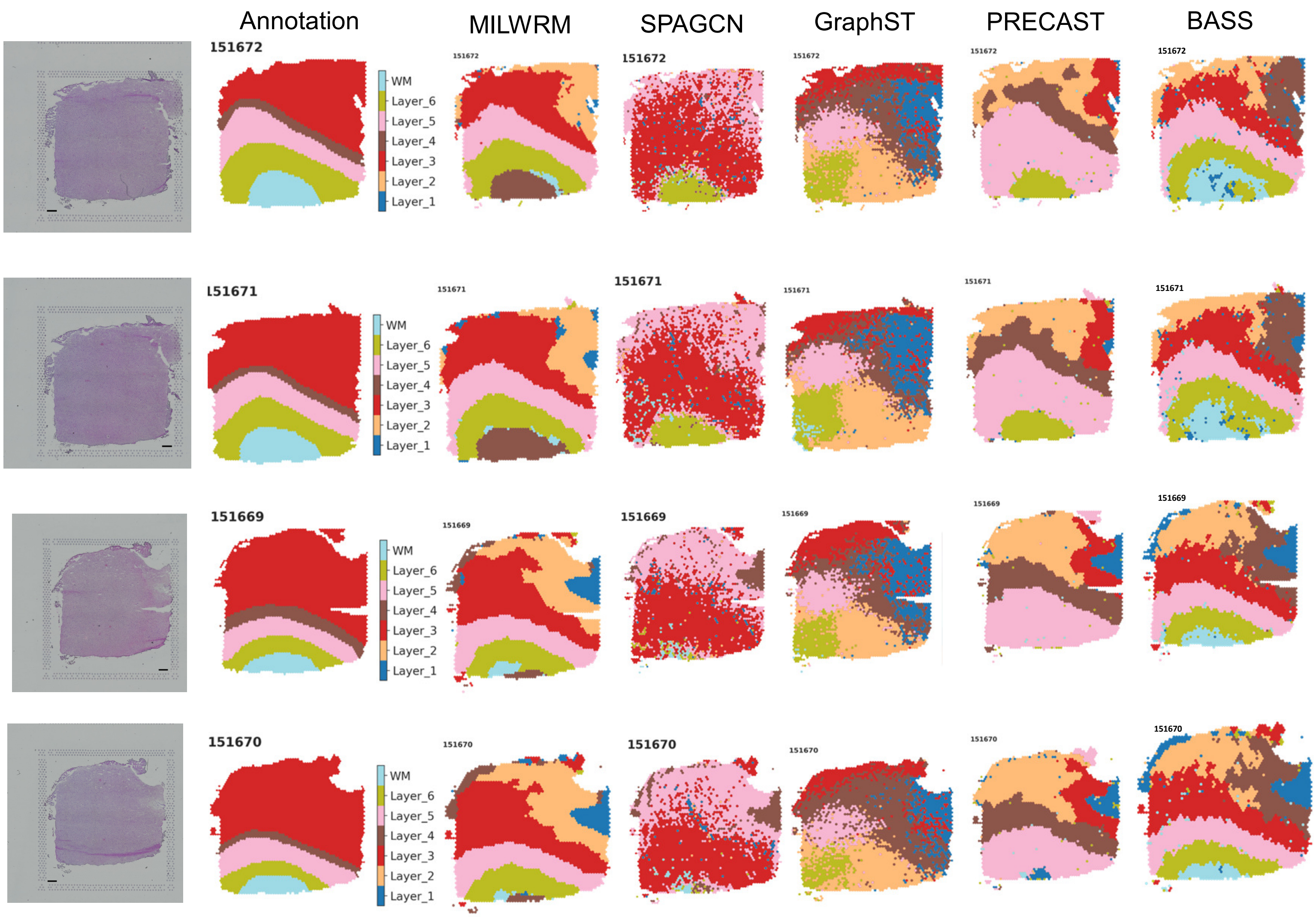

Figure S7. Tissue domains for DLPFC sections (151669-151672). MILWRM, SpaGCN, GraphST, PRECAST and BASS detected tissue domains (*k*=7) overlaid on DLPFC specimens (scale bar = 500 µm).

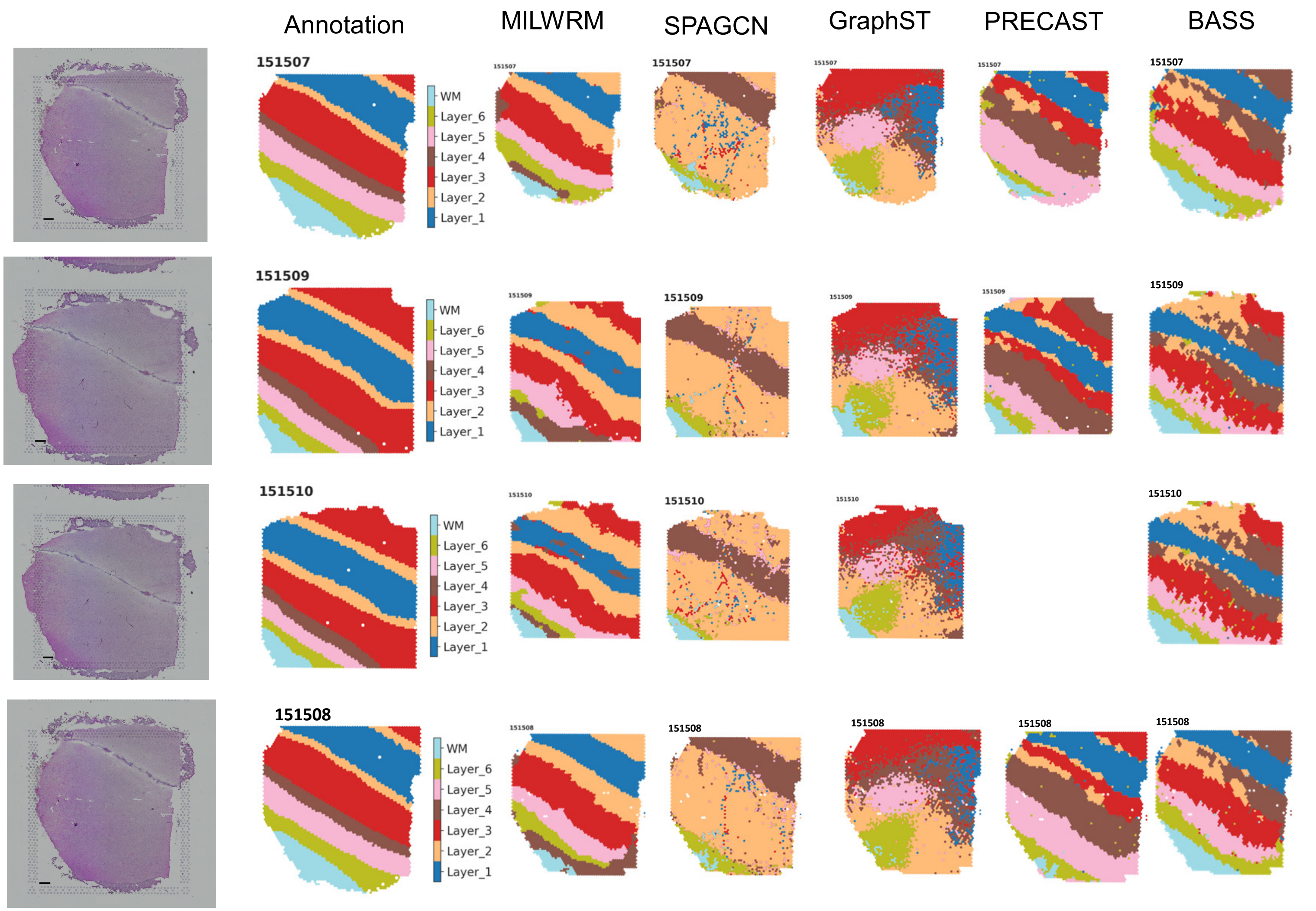

Figure S8. Tissue domains for DLPFC sections (151507-151510). MILWRM, SpaGCN, GraphST, PRECAST and BASS detected tissue domains (*k*=7) overlaid on DLPFC specimens. PRECAST caused some unexpected trimming in 151510 (scale bar = 500 μm).

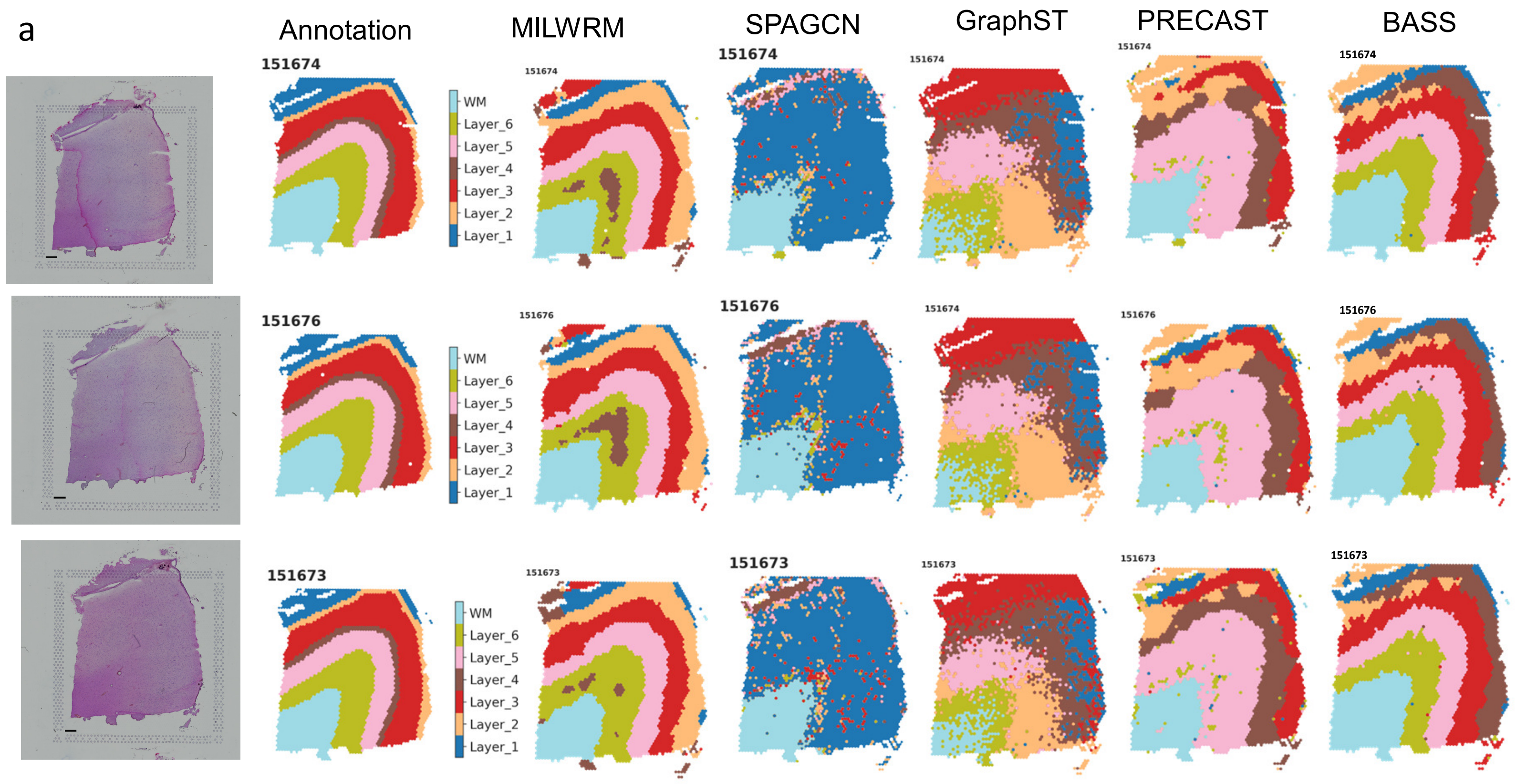

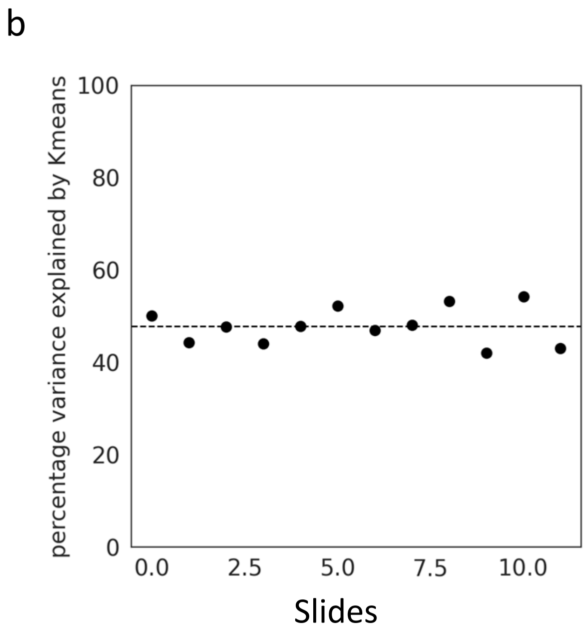

Figure S9. Tissue domains for DLPFC sections (151673,151674 and 151675). (a) MILWRM, SpaGCN, GraphST, PRECAST and BASS detected tissue domains (*k*=7) overlaid on DLPFC specimens (scale bar = 500 μm). (b) Scatter plot for percentage variance explained by the *k*-means model over 12 samples with each dot corresponding to a sample; dotted line is mean variance explained across all samples.

Supplementary Table 1. Summary table for human colonic sample metadata

Batch name	Slide region	Tissue category	Broad precancer type
HTA11_10167_0000_01_01	HTA11_10167_0000_01_01_region_001	normal	SSL
HTA11_10167_0000_01_01	HTA11_10167_0000_01_01_region_002	normal	SSL
HTA11_10167_0000_01_01	HTA11_10167_0000_01_01_region_003	Tumor	SSL
HTA11_10623_0000_01_01	HTA11_10623_0000_01_01_region_001	normal	AD
HTA11_10623_0000_01_01	HTA11_10623_0000_01_01_region_002	normal	AD
HTA11_10623_0000_01_01	HTA11_10623_0000_01_01_region_003	normal	AD
HTA11_10623_0000_01_01	HTA11_10623_0000_01_01_region_004	Tumor	AD
HTA11_10623_0000_01_01	HTA11_10623_0000_01_01_region_005	normal	AD
HTA11_10623_0000_01_01	HTA11_10623_0000_01_01_region_006	normal	AD
HTA11_10711_0000_01_01	HTA11_10711_0000_01_01_region_001	Tumor	AD
HTA11_4255_0000_02_02	HTA11_4255_0000_02_02_region_001	Tumor	SSL
HTA11_4255_0000_02_02	HTA11_4255_0000_02_02_region_002	normal	SSL
HTA11_6298_0000_04A_03	HTA11_6298_0000_04A_03_region_001	Tumor	AD
HTA11_6298_0000_04A_03	HTA11_6298_0000_04A_03_region_002	Tumor	AD
HTA11_6298_0000_04A_03	HTA11_6298_0000_04A_03_region_003	normal	AD
HTA11_6801_0000_01_01	HTA11_6801_0000_01_01_region_001	Tumor	SSL
HTA11_7179_0000_02_02	HTA11_7179_0000_02_02_region_001	Tumor	AD
HTA11_7862_0000_02_02	HTA11_7862_0000_02_02_region_001	Tumor	AD
HTA11_7862_0000_02_02	HTA11_7862_0000_02_02_region_002	normal	AD
HTA11_7956_0000_02_05	HTA11_7956_0000_02_05_region_001	normal	SSL
HTA11_7956_0000_02_05	HTA11_7956_0000_02_05_region_002	Tumor	SSL
HTA11_7956_0000_02_05	HTA11_7956_0000_02_05_region_003	Tumor	SSL
HTA11_8099_0000_02_01	HTA11_8099_0000_02_01_region_001	normal	SSL
HTA11_8099_0000_02_01	HTA11_8099_0000_02_01_region_002	normal	SSL
HTA11_8099_0000_02_01	HTA11_8099_0000_02_01_region_003	normal	SSL
HTA11_8099_0000_02_01	HTA11_8099_0000_02_01_region_004	normal	SSL
HTA11_8099_0000_02_01	HTA11_8099_0000_02_01_region_005	Tumor	SSL
HTA11_8099_0000_02_01	HTA11_8099_0000_02_01_region_006	normal	SSL
HTA11_8622_0000_01E_01	HTA11_8622_0000_01E_01_region_001	normal	SSL
HTA11_8622_0000_01E_01	HTA11_8622_0000_01E_01_region_002	Tumor	SSL
HTA11_8622_0000_01E_01	HTA11_8622_0000_01E_01_region_003	Tumor	SSL
HTA11_8622_0000_01E_01	HTA11_8622_0000_01E_01_region_004	Tumor	SSL
HTA11_8622_0000_01E_01	HTA11_8622_0000_01E_01_region_005	normal	SSL
HTA11_866_0000_02_03	HTA11_866_0000_02_03_region_001	Tumor	AD
HTA11_8920_0000_02_02	HTA11_8920_0000_02_02_region_001	Tumor	SSL
HTA11_9341_0000_01A_01	HTA11_9341_0000_01A_01_region_001	Tumor	SSL
HTA11_9408_0000_02A_05	HTA11_9408_0000_02A_05_region_001	Tumor	AD
HTA11_9408_0000_02A_05	HTA11_9408_0000_02A_05_region_002	Tumor	AD

Supplementary Table 2. Coefficient summary table for association between size of tissue domain and pre-cancer subtype

Pixel size of tissue domain	Estimate	SE	Chi-square	p-value	RESI	p_{FDR}
Differentiated tissue domain	0.412	2.12e-01	3.774	0.052	0.430	0.130
Pericryptal Stroma	0.240	1.31e-01	3.366	0.067	0.397	0.133
Smooth Muscle	-0.341	2.83e-01	1.449	0.229	0.173	0.381
Mucus	0.036	1.68e-01	0.046	0.830	0.000	0.922
Deep Lamina Propria	0.010	2.10e-01	0.002	0.962	0.000	0.962
Abnormal Layer	0.996	4.68e-01	4.534	0.033	0.485	0.130
Proximal Lamina Propria	0.145	1.87e-01	0.604	0.437	0.000	0.581
Crypt Lumen	-1.116	5.65e-01	3.904	0.048	0.440	0.130
Stem Layer	-0.680	1.48e-01	21.220	0.000	1.161	0.000
Total	50593.575	6.92e+04	0.535	0.464	0.000	0.581

Supplementary Table 3. Coefficient summary table for association between size of maximum connected component of each tissue domain and pre-cancer type

Maximum pixel size of connected components in tissue domain	Estimate	SE	Chi-square	p-value	RESI	p_{FDR}
Differentiated tissue domain	0.045	0.178	0.065	0.798	0.000	0.898
Pericryptal Stroma	0.012	0.136	0.008	0.929	0.000	0.929
Smooth Muscle	-0.164	0.144	1.299	0.254	0.141	0.357
Mucus	0.535	0.105	25.849	0.000	1.287	0.000
Deep Lamina Propria	0.114	0.084	1.836	0.175	0.236	0.357
Abnormal Layer	0.275	0.254	1.177	0.278	0.109	0.357
Proximal Lamina Propria	-0.142	0.112	1.595	0.207	0.199	0.357
Crypt Lumen	-0.262	0.193	1.850	0.174	0.238	0.357
Stem Layer	-0.435	0.175	6.164	0.013	0.587	0.059
